# Chronic Non-cancer Pain Management and Addiction: A Review

**DOI:** 10.7759/cureus.6963

**Published:** 2020-02-12

**Authors:** Alessia Genova, Olivia Dix, Mala Thakur, Pritpal S Sangha

**Affiliations:** 1 Medicine, Xavier University School of Medicine, Oranjestad, ABW; 2 Medicine, Medical University of Lublin, Lublin, POL

**Keywords:** pain management, opioids, non-cancer, addiction, tolerance, chronic pain

## Abstract

The use of opioids in chronic non-cancer pain (CNCP) has been a fast-rising treatment phenomenon in the last two decades. Though opioids are advocated in various chronic pain management guidelines, their use in chronic non-cancer pain still remains controversial as evidence of this approach is still weak. This paper highlights potential adverse effects associated with opioid use in pain management, including an increase in tolerance, dependence, and addiction outcomes. Nonetheless, opioids have an important place in contemporary CNCP management for selected patients. However, pain management must involve regular monitoring and the use of a multimodal pain management strategy. It is essential that the treating physician must be up-to-date with the form of therapy so that they have the requisite confidence to prescribe opioids and effectively manage adverse effects. Moreover, the adverse effects should be treated promptly to enhance patient compliance. Since this approach is beneficial for some patients, opioid treatment should only be applied on a case by case basis.

## Introduction and background

Over the last decades, the use of opioids in cancer and chronic non-cancer pain in the United States of America has increased significantly. The World Health Organization (WHO) considers opioids as an important analgesic ladder for various types of pain. However, in the case of chronic non-cancer pain, such as neuropathic pain, they are used on a case by case basis [1]. Generally, the use of opioids in pain management is supported by multiple randomized trials that have demonstrated high-quality evidence for their use in the short-term basis and in low doses. Opioids are also included as second or third-line treatment under several international guidelines. Nevertheless, a significant number of epidemiological studies demonstrated that there was minimal change in pain outcomes or quality of life of patients who used these drugs for a long period [2]. Furthermore, prescription and use of strong opioids for long periods increased the risk of serious adverse effects, including overdose, addiction, and death. In this case, patient selection and outcome assessments for long-term use are very critical going forward. In this regard, this paper intends to provide in-depth insight into the opioid role in chronic non-cancer pain management including addiction and other adverse effects.

Opioid crisis in America

Misuse of opioids is a major public health concern in the United States as the prevalence of misuse has been consistently rising since the late 1990s resulting in thousands of opioid-related deaths. According to a 2015 National Survey on Drug Use and Health, 91.8 million adults in the United States were found to use prescription opioids [2]. Among them, 11.5 million misused the drugs while 1.9 million had developed substance use disorder. In this survey, the respondents indicated that their main motivation for the misuse of the drugs was physical pain relief. In addition, it was found that people who were socioeconomically disadvantaged or had behavioral health problems were at a greater risk of misusing drugs. Furthermore, the survey indicated that 59.9 percent of the respondents had used opioids without prescription while 40.8 percent obtained free prescriptions from family and friends. In another study that examined opioid use disorders, it was found that people who did not obtain prescriptions started opioid use at a relatively early stage where they obtain the drugs illicitly for recreational purposes [3].

According to another study, opioid misuse was the primary cause of overdose and death. In the United States, substance overdose resulted in 52,404 deaths in 2015 with more than 33,000 being attributed to the use of opioids [4]. Though prescription opioids are blamed for this prevalence, in reality, prescribed opioids had declined significantly since 2010. This shows that the current opioid crisis can be attributed to heroin or illegally manufactured fentanyl. Since prescription opioids have become less available, heroin has become more frequent and with imprecise dosing and limited tolerance in new users, it has resulted in considerable fatalities [1]. Benzodiazepines are another psychoactive substance that are often linked to substance misuse deaths. However, in the last 25 years, medical practitioners have become increasingly willing to prescribe opioids for chronic non-cancer pain. This increase in medicinal opioid use has also been linked to an increase in mortality. In the United States, deaths attributed to opioid analgesics increased from 4,041 in 1999 to 14,459 in 2007. Currently, deaths associated with opioid misuse and use is more common than multiple myeloma, HIV, and alcoholic liver disease. Though drug-specific data are not universally available, the use of oxycodone is linked to deaths in jurisdictions, such as Ontario, Canada, and Victoria, Australia [5].

The struggle of chronic non-cancer pain

Chronic non-cancer pain (CNCP) can be typically described as moderate or severe pain that lasts for six or more months and is attributed to conditions such as neuropathic pain, rheumatoid arthritis, lower back pain, osteoarthritis, fibromyalgia and a range of several other conditions [2]. People with CNCP rarely enjoy their lives as they find it hard to sleep, often feel fatigued and depressed and struggle to form meaningful social relationships with family and friends. In the United States, 26 percent of adults suffer from at least one chronic condition, such as arthritis, diabetes and hypertension. Most of the time, these people experience persistent and recurrent pain that makes their conditions even worse. As a result, CNCP is one of the main causes of morbidity and interference with the person’s ability to perform their day to day activities. Chronic conditions not only affect the patient but also their families, communities and the health systems, as they are also stakeholders in the struggle to control the disease and its complications. These continuous and endless struggles contribute to frustrations and depression among these patients. Frustration among these patients is linked to the complexity and unpredictability of pain. As a result, patients have been found to stop their self-care activities as well as essential prescriptions. In other cases, the suffering person may lose interest in living and would prefer to die other than continue with the present living situation [4]. In this regard, opioids are used to relax these patients and relieve some of the pain they feel.

## Review

Method

To address the issue of opioid use in CNCP management, we searched the internet for terms: addiction, chronic pain, opioid dependence, chronic non-cancer pain management, and substance dependence. The search was confined to peer-reviewed papers that talked about opioid use in the United States of America. Furthermore, only papers that dealt with CNCP were considered. As a result, six papers were found to comprehensively address the treatment of CNCP with opioids while four papers dealt with primary opioid addiction and tolerance.

Discussion

Opioid Use in the Management of Chronic Non-cancer Pain

The goal of CNCP treatment is similar to the treatment of all other chronic pain patients and that is to improve pain outcomes and maintain functionality. Some of the targeted milestones in successful pain management strategy include positive coping skills, stress minimization and establishment of better social support systems. Effective management of comorbid neuropsychiatric complications is also to maximize functionality [1]. In the case of opioid use, the physician must also be aware of the drug addiction potential. Opioids are largely used in CNCP management as it is believed that they provide sufficient pain relief. However, studies indicated that the use of strong opioids could potentially lead to adverse effects, such as respiratory depression and reduced levels of consciousness thus the need to only prescribe minimal levels of opioids [4]. Furthermore, though some opioids, such as methadone and buprenorphine can also be used to treat pain, the duration of analgesic action associated with them is often shorter compared to the withdrawal and craving symptoms. In this regard, it is recommended that they be administered more frequently [5].

Meanwhile, in the case of patients who are in opioid substitution therapy, one must be careful of the degree of opioid analgesic tolerance thus they may need increased levels of dosage to appreciate pain relief. In 2013, Chang and Compton indicated that in the case of methadone maintenance patients, they often experience higher pain sensitivity and would therefore need higher opioid analgesic requirement [3]. Depending on the type of the substance that the patient previously used or misused, exposure to psychoactive medications can sometimes lead to relapse. In this case, clinicians must assess the patient’s probability of relapse when using opioids in pain management on patients who had been treated for addiction before. Although addiction can be effectively managed for CNCP using opioids, the goal of their treatment must also include preventing exacerbation of the substance use disorder (SUD). In such cases, relapse prevention strategies should form an integral part of the plan of care [2]. In these instances, before prescribing opioids, the clinician should assess the patient for intrapersonal and interpersonal stressors of SUD.

Tolerance Versus Addiction

Drug addiction, dependence, and tolerance affect the body and brain of a person in a different manner. Tolerance refers to a situation where a person is no longer responding to a drug. In this case, a higher dose of the drug will be required to achieve a similar effect than when the drug was first used. This is the same thing that happens when a person is suffering from SUD. Tolerance can be classified into acute, chronic and learned tolerance [1]. Acute tolerance results from repeated exposure to the drug over a relatively short period of time. Chronic tolerance is caused by a person’s adaptation to constant drug exposure over weeks or months. Learned tolerance is attributed to frequent exposure to certain drugs. However, tolerance often disappears when the act of exposure is considerably altered [6]. On the other hand, dependence refers to a case where the person cannot stop using the drug as the body will go through withdrawal [4]. Withdrawal effects may include physical and mental symptoms ranging from mild to life-threatening ones. When a person takes prescription drugs on a daily basis over a long period of time, they need to go off the drug gradually to minimize or avoid withdrawal effects. However, dependence does not necessarily mean addiction. Unlike dependence and tolerance, addiction is considered a disease since an addicted person cannot stop taking the drug despite the negative consequences associated with the drug. In this case, a person can be dependent or have a high tolerance to the drug without necessarily being addicted.

Chronic pain and addiction can be conceptualized as a syndrome. For instance, some studies indicated that pain is one of the contributing factors to addiction. Many scholars hypothesize that untreated pain is a significant risk factor for relapse in individuals with addiction in remission [7,8]. These studies also suggested that exposure to opioids among chronic pain patients with SUD history enhanced their risk of opioid abuse and potential relapse. Physiological and psychological features of addiction thus make pain relatively difficult to manage or treat. When a patient chronically uses opioid drugs, their ability to process pain stimuli through sympathetic stimulation, hypothalamic-pituitary-adrenal axis dysregulation, and proinflammatory immune-system activation will be adversely affected leading to increased pain sensitivity and reduction in pain tolerance [9]. The effect of chronic use of opioids, therefore, results in the reorganization of nociceptive pathways in the brain causing an increase in pain perception. Nevertheless, pain patients with active addiction were found to be less likely to respond to non-opioid pain treatment strategies, such as physical and behavioral interventions. However, one must recognize that self-medication use opioids can also become a coping mechanism for CNCP patients leading to misuse of prescription opioids.

Successful Chronic Pain Management in the Recovering Addicted Patient

There are several options that various studies suggested for chronic pain treatment. These approaches can be categorized as physical medicine, behavioral medicine, neuromodulation, pharmacologic, interventional, and surgical approaches [3]. For optimal patients’ outcomes, multiple approaches coordinated by a multidisciplinary team should be implemented. Collaborative care models were found to improve pain management and patient outcomes. This implies that a successful model should not focus solely on medication but should include other treatment modalities to meet treatment goals. Furthermore, patients with chronic pain require regular evaluation, education, and reassurance, as well as setting reasonable expectations for response [8]. The collaborative model was also found to improve the patient's quality of life, both physically and emotionally. The collaborative model also offers flexibility that allows other models to be incorporated, thus enhancing the effectiveness of pain management strategy, especially when using opioids.

To effectively manage CNCP using opioids, clinicians are encouraged to adopt practices that will prevent SUDs through assessment of risks and providing pain care. The clinicians must also differentiate patients with SUD from those who are at a higher risk of developing SUD. Though no universal guidelines exist for direct management of pain using opioids, studies indicate that adherence to the clinician’s guidelines is one of the core components of effectiveness [6]. In this case, pain management guidelines should be flexible to allow for modification based on new evidence and the level of exposure of the patients and his/her family to exposure. In 2003, Stannard and Johnsonindicated that the use of codeine and morphine contributed more positively to pain relief than other treatment approaches [8]. Even in the case of neuropathic pain, which is considerably opioid resistant, the studies found that the body was not completely unresponsive to opioid administration [8]. Besides, the use of opioids in pain management has been criticized due to the addictive potential of these drugs. However, clinicians insist that carefully prescribed opioid administration cannot lead to addiction. Moreover, experts believe that opioid treatment does not carry a significant risk of iatrogenic addiction.

Additionally, non-drug interventions are also encouraged in some cases though the evidence of their efficacy is also still limited. For example, psychological therapies could lead to modest positive outcomes when administered by trained and experienced staff. The lack of universally proven evidence-based criteria for CNCP diagnosis and management has led to the suggestion of various criteria. First, a physiological criterion is suggested for long term opioid treatment. These criteria should be recommended based on the tolerance and withdrawal potential on the patient [10]. This criterion is based on the knowledge that chronic pain is a complex interaction between physiological and psychosocial factors, thus a successful intervention must involve the two therapeutic disciplines. Here, a clinical psychologist and physical therapist work together with the patient’s support systems, such as family, nurse, and counselors to establish and implement the most effective treatment intervention for the patient. The goal of this approach is to minimize the physiological and psychological symptoms of chronic pain by decreasing pain intensity, increasing the patient’s physical activity, controlling the management of pain medication, facilitating return to work and reducing the use of health-care services. This approach also recognizes that patients also undergo emotional distress, anxiety and irritability [7]. In this case, a cognitive-behavioral intervention is designed to assist the patient to control their adverse emotional reactions to chronic pain through therapy sessions that intend to help him/her to identify maladaptive and negative thoughts, dispute irrational thinking, construct and repeat positive self-statements, learn distraction techniques and improve social support.

Second, the behavioral criterion is recommended for a chronic pain patient of a therapeutic dependence. Patients of therapeutic dependence are more anxious to secure supplies of analgesics as they fear that running out would worsen their condition. However, one must appreciate that the very nature of chronic pain can decrease a person’s desire and ability to socialize while those who frequently visit medical practitioners can give a false image of addiction. Literature acknowledges that cognitive-behavioral technique (CBT) is effective in minimizing pain compared to other independent therapies [9]. However, it must be used alongside other physiological interventions as they do not directly affect pain but focus on improving mood and alleviating anxiety. Another concern raised regarding the use of opioids in CNCP is whether it can be applied in the case of patients who are suffering from opioid use disorder. In all cases, clinicians must be cautious what their patients report, given that some of them may exaggerate their pain in order to obtain opioids due to the drug’s psychoactive properties. In the meantime, clinicians who may ‘over-prescribe’ opioids should be considered dishonest and unethical. In the case of patients with opioid use disorder, methadone was found to be relatively effective in the management of comorbid opioid dependence and chronic pain [7]. Before treatment strategy is decided, it must be noted that long-term opioid use can lead to more challenging problems, therefore good evidence is needed before its implementation as the sole pain management strategy. One must also note that there is no single pain treatment drug that works for all people. In this regard, the prescription of opioids for neuropathic pain should be minimized.

Multidisciplinary Approach to Chronic Non-cancer Pain

Chronic non-cancer pain affects the quality of life and socio-economic activities of millions of Americans. These pains also lead to the expenditure of billions of dollars annually and loss of productivity in the economy. Though there is no definitive cure for chronic non-cancer pain, many studies suggest a multidisciplinary pain program that emphasizes ensuring that the person’s independence is restored and their quality of life improved [3]. Usually, multidisciplinary pain management approaches are developed along with a bio-psycho-social model of chronic non-cancer pain. Multidisciplinary care is essentially transformative care as it incorporates comprehensive, patient-centered self-management strategies with evidence-based treatments as routine care for various chronic pain conditions and ensures that consequences are effectively prevented and managed. This kind of transformative care significantly improves long-term outcomes and reduces the dependence of the patient on the health care system, thereby leading to improvement of both the patient’s life and health care system. For this reason, health leaders should always strive for the integration of self-management strategies into a clinical practice that aims at engaging, educating and empowering people to prevent chronic pain and addiction [10]. In this regard, transformative care assists in improving the experience of the patient with care, improving patient health, and controlling healthcare costs.

The integration of training with treatment is another important component of multidisciplinary pain management. Training of health professionals can help to minimize some of the barriers that may reduce the effectiveness of various strategies. Some of the challenges that can be solved through training include care coordination and fragmentation, poor communication and conflicting treatments. McCrorie et al. indicated that the approach is relatively effective in the treatment of chronic back pain and has been highly recommended for the treatment of low back pain [9]. Some of the common features of this model include standardized group sizes and treatment strategies and high intensity of treatment. In relation to treatment strategy, the approach consists of interventional injection techniques, such as epidural, periradicular and facet joint injections, which are done frequently. These injections are augmented with other treatment approaches, including modification of analgesic medication, transcutaneous electrical nerve stimulation, aquatraining, massage therapy, and back education, among others [10].

The multidisciplinary approach also encompasses behavioral management. Physiotherapists perform behavioral therapy at least twice a week on the patient based on the severity of the CNCP as well as other psychological cofactors. According to studies based on the Numeric Rating Scale (NRS) and the Oswestry Disability Index (ODI), behavioral management strategies were found to improve pain outcomes and functionality of patients. The study indicated that the integration of multiple strategies with different components in pain management was found to lead to better results than when a single intervention was applied [9]. The efficacy of the multidisciplinary programs was found to be better than the standard medical treatment and other non-multidisciplinary treatments. Moreover, multidisciplinary programs for chronic pain patients allow them to access specialized treatment. Nonetheless, as with any other intervention, multidisciplinary approaches are affected by both methodological and conceptual limitations which make it relatively difficult to implement.

## Conclusions

Opioids play an important role in the management of chronic non-cancer pain. In this regard, this paper intended to provide in-depth insight into CNCP management using opioids and its adverse effects. It also highlighted various concerns with acute pain and palliative care, especially in relation to opioid dependence and addiction. Due to these concerns, we have suggested that opioids should not be the drug of choice for chronic pain and should only be used when necessary. Hence, a clinician’s decision to initiate opioid-based pain treatment should be evidence-based and informed by accurate long-term efficacy outcomes and potential adverse effects on the patient. It is evident that opioids treatment can be beneficial to some patients, therefore, response should be analyzed on a case by case basis. However, a comprehensive conclusion on opioid use in pain management cannot be made as the literature did not offer satisfactory evidence. Therefore, it is important that the approach selected for every person should be able to offer maximum benefits and protect them from adverse risks. Accordingly, there is still a need for further research, especially in relation to opioid treatment, its efficacy and addiction potential. Finally, this paper suggests that the clinician should always treat pain complaints from patients with opioid use disorder with skepticism.
